# Commentary: Piéron's law is not just an artifact of the response mechanism

**DOI:** 10.3389/fphys.2015.00190

**Published:** 2015-06-30

**Authors:** José M. Medina, José A. Díaz

**Affiliations:** Departamento de Óptica, Facultad de Ciencias, Edificio Mecenas, Universidad de GranadaGranada, Spain

**Keywords:** human reaction time, power laws, decision making, information entropy, statistical physics, fractals

It has long been known that the mean human reaction/response time (RT), *t_RT_*, decreases as the stimulus strength or intensity *S* increases (Cattell, 1886), reaching an asymptotic value or plateau, *t*_*RT*_0__, at very high *S*-values in all sensory modalities. A well-established power law, namely, Piéron's law, describes mathematically that empirical relationship (Piéron, 1914; Luce, 1986):

(1) $$t_{RT}=t_{RT_{0}}+kS^{-p}$$

where *k* and *p* are coefficients; the latter being a fractional exponent that controls the *RT* decay. Donkin and van Maanen has investigated the origin of Piéron's law based on a version of the Linear Ballistic Accumulator model. They concluded that Piéron's law is not due only to a decision making process. Various types of models have been proposed for describing the foundations of Piéron's law (Link, 1992; Baird, 1997; Stafford and Gurney, 2004; Hsu, 2005; Palmer et al., 2005; Stafford et al., 2011; Servant et al., 2014; Verdonck and Tuerlinckx, 2014). The model proposed by Donkin and van Maanen belongs to an influential class of models in mathematical psychology, i.e., sequential sampling models. In general, these models of Piéron's law assume the existence of an internal variable threshold. From the stimulus onset, there is an accumulation of noisy “sensory information” or “evidence” until a response criterion is reached. However, the concept of information is not properly defined within the context of information theory and plays no role. A decisional stage is usually implemented in the form of random walk, diffusion, and accumulator models. Despite these models mimic the functional form of Piéron's law, it is not clear whether they are able to explain the internal structure of *k* and *t*_*RT*_0__ in Equation (1) and to provide more detailed predictions based on threshold mechanisms. For instance, all these models often postulate that the asymptotic term *t*_*RT*_0__ is nearly invariant and includes non-decision components (e.g., the motor execution time) that do not hold a chronological order. However, *k* and *t*_*RT*_0__ span a range of experimental values and depend on early sensory processing (Pins and Bonnet, 1997; Plainis and Murray, 2000; Murray and Plainis, 2003).

There is an information-theoretic approach, which is rarely mentioned in the literature of Piéron's law, that derives Equation (1) from an optimal information process in sensory perception. In this framework, the first stage of RTs always corresponds to an efficient stimulus encoder. Only after this initial stage there is a *bona fide* accumulation of information over time, Δ*H* > 0 (e.g., measured in bits), that is related with power law behavior at the threshold, βS^p^_0_. *S*_0_, and β indicate an internal threshold and a normalization coefficient, respectively. Piéron's law results from a temporal sequence of events that differentiates those components near the threshold *S*_0_ from those at suprathreshold conditions (S > S_0_). The coefficient *k* follows a power law (Norwich et al., 1989; Norwich, 1993):

(2) $$k=t_{RT_{0}}S_{0}^{p}$$

The asymptotic term *t*_*RT*_0__ only contains the initial encoding time *t*_0_ and βS^p^_0_, and it obeys a similar power law (Medina, 2012):

(3) $$t_{RT_{0}}=t_{0}\left(1+\beta S_{0}^{p}\right)$$

Equation (2) corroborates that the coefficient *k* has a direct link with a threshold mechanism in human vision (Plainis and Murray, 2000; Murray and Plainis, 2003; Medina and Diaz, 2005, 2006).

There is a chronological order that cannot be violated, namely, *t*_RT_ > t_RT_0__ > t_0_ > 0. This is a direct consequence of Δ*H* and involves the principle of causality over time, which states that the effect cannot be before the cause. The formation of a threshold at *t*_*RT*_0__ cannot precede the stimulus encoding at *t*_0_, and those processes at suprathreshold conditions at *t_RT_* cannot precede those at *t*_*RT*_0__ either (Medina et al., 2014). Further, Piéron's law is shape-invariant under rescaling (Chater and Brown, 1999) in a fractal-like process. In the rate domain (1/RT), Piéron's law has a direct link with the Naka-Rushton equation in neurophysiology (Naka and Rushton, 1966; Carandini and Heeger, 2012). Let, *R* = 1/*t_RT_*, and *R_M_* = 1/*t*_*RT*_0__, from Equations (1) and (2) (Medina, 2009):

(4) $$R=\frac{R_{M}}{1+{\left(\frac{S_{0}}{S}\right)}^{p}}$$

Equations (2) and (3) show that threshold impairment in *S*_0_ leads to longer RTs and consequently, it modifies Piéron's law in Equation (1). We exemplify the non-trivial effects of anomalous power law behavior βS^p^_0_ in Piéron's law in two different scenarios. *β*, *S*_0_, and *p* could vary based on several experimental factors. Similar examples follow in the same way. In the first example, we illustrate Piéron's law in amblyopia. Amblyopia (usually called “lazy eye”) affects approximately 3% of human population and is a combination of visual deficits that impairs binocular vision from physiological alterations during early development (Ciuffreda et al., 1991; Howard, 2002). Figure 1A simulates the typical variation of the reciprocal of *S*_0_ for spatial sine-wave gratings in normal and amblyopic vision. Threshold values *S*_0_ are higher in the amblyopic eye at high spatial frequencies (Ciuffreda et al., 1991). This deficit is the principal responsible for higher βS^p^_0_, *k*, and *t*_*RT*_0__ values in Equation (1) and consequently, for longer RTs in amblyopic vision (Figure 1B) (Pianta and Kalloniatis, 1998). In the rate domain (Equation 4), amblyopic vision is limited because it gives saturated responses sooner (Figure 1C).

**Figure 1:**
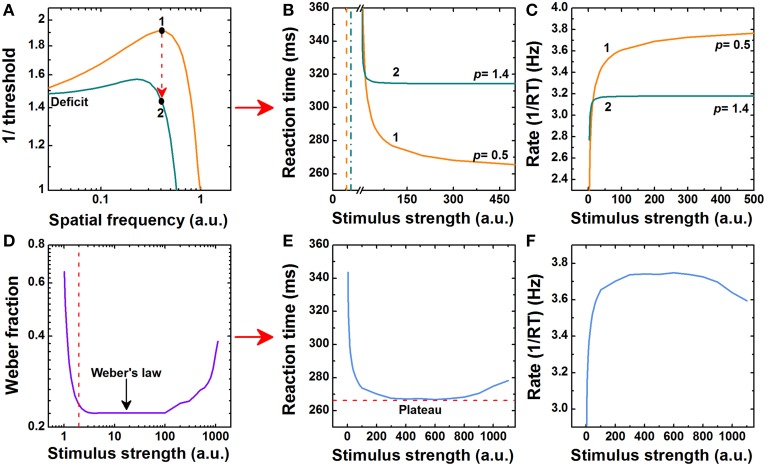
**Examples of Piéron's law**. **(A)** Double logarithmic plot of the contrast sensitivity function (1/threshold) as a function of the spatial frequency for sine-wave gratings. Points labeled as “1” and “2” indicate two threshold values at the same spatial frequency that correspond to normal and amblyopic vision, respectively (Ciuffreda et al., 1991). In both cases the adapting conditions were the same and fixed. **(B)** Linear plot of Piéron's law as a function of the stimulus strength *S*. Vertical dash and dash-dot lines represent those threshold values labeled as “1” and “2” in **(A)**, respectively. Solid lines labeled as “1” and “2” show the corresponding Piéron's law in normal and amblyopic vision, respectively (Pianta and Kalloniatis, 1998). Simulation parameters in normal vision: *t*_0_ = 44, β = 294.39, *S*_0_ = 0.52, *p* = 0.5. Amblyopic eye: *t*_0_ = 44, β = 439.35, *S*_0_ = 0.71, *p* = 1.4. **(C)** Linear plot of Piéron's law in the rate domain (1/RT). Solid lines labeled as “1” and “2” follow the same as in **(B)**. **(D)** Double logarithmic plot of the Weber fraction (Δ*S/S*) as a function of intensity *S*. Vertical dash line separates the Rose-de Vries regime at low *S*-values from Weber's law. **(E)** Linear plot of Piéron's law as a function of the stimulus strength *S*. The adapting conditions now vary. The power law βS^p^_0_ is mapped onto a Weber fraction-type power law (Δ*S/S*)*^p^* (Medina, 2011) and was varied from Weber's law to the terminal rise in **(D)**. Simulation parameters: *t*_0_ = 150, β = 1, *p* = 0.33. **(F)** Linear plot of Piéron's law in the rate domain. (a.u.) = arbitrary units.

The second example illustrates the van der Mollen-Keuss effect in RTs. The van der Mollen-Keuss effect imposes a limitation to Piéron's law by producing a U-shaped function at very high *S*-values (van der Molen and Orlebeke, 1980; Jaśkowski and Włodarczyk, 2006; Marino and Munoz, 2009). βS^p^_0_ also depends on the sensory adaptation level (Plainis and Murray, 2000; Murray and Plainis, 2003; Medina, 2011). Figure 1D simulates the differential threshold relative to the background or Weber fraction (Δ*S/S*) as a function of the intensity *S*. The minimum value corresponds to Weber's law. There is a terminal rise at very high intensities. By interpreting the βS^p^_0_ as a Weber fraction, the terminal rise in Weber's law which is observed in many modalities gives rise to an abrupt increment in both *k*, and *t*_*RT*_0__ in Equation (1) for high intensities. The van der Mollen-Keuss effect can therefore be explained theoretically as a consecuence of an entropy-based approach together with Weber's law (Figure 1E). In the rate domain (Equation 4), the reciprocal of RT shows an inverted U-shaped function (Figure 1F). This suggests a correlation with specific neural activity (Peirce, 2007). Outside the framework of Piéron's law, a more ellaborate approach to the Weber fraction and Weber's law has been developed using the same information-theoretic formalism (Norwich, 1993; Norwich and Wong, 1997).

Donkin and van Maanen fitted three different experimental data sets to test the validity of their assumptions (Donkin and van Maanen, 2014). Good fits to experimental data are neccesary but insufficient to support theoretical models with free parameters. Power laws in complex systems are better supported by models that constraint possible results and predict how experiments agree with such constraints (Roberts and Pashler, 2000; Kello et al., 2010; Stumpf and Porter, 2012). Hence, we have introduced a poweful approach for analyzing the relationship between an internal variable sensory threshold and Piéron's law by using information theory and power law scaling.

## Conflict of interest statement

The authors declare that the research was conducted in the absence of any commercial or financial relationships that could be construed as a potential conflict of interest.
